# A 33-year-old patient with human immunodeficiency virus on antiretroviral therapy with efavirenz-induced complex partial seizures: a case report

**DOI:** 10.1186/s13256-016-0876-9

**Published:** 2016-04-13

**Authors:** Nathan Yakubu Shehu, Victor Ojeh, Godwin Osaigbovo, Patricia Agaba, Oche Agbaji

**Affiliations:** 1 Jos University Teaching Hospital, Jos, Plateau State Nigeria; 2 University of Jos, Jos, Plateau State Nigeria

**Keywords:** Efavirenz, Seizures, HIV

## Abstract

**Background:**

Efavirenz is a commonly prescribed antiretroviral drug that is largely well tolerated. However, seizure disorder is a rare side effect. Prompt identification and immediate replacement of efavirenz with an alternative drug would effectively stop the seizures. To the best of our knowledge, we present the first reported case in the literature of complex partial seizures arising due to efavirenz.

**Case presentation:**

We report a case of a 33-year-old Nigerian man treated with an efavirenz-based antiretroviral regimen for human immunodeficiency virus infection. He presented with seizures soon after commencement of antiretroviral drugs. His magnetic resonance imaging results were unremarkable. His blood levels of sodium, glucose, urea, and creatinine were within normal limits. However, his electroencephalogram showed intermittent bursts of high-voltage sharp waves and spikes bilaterally over frontotemporoparietal regions, a finding consistent with complex partial seizures. His efavirenz plasma level was 209.55 μg/ml. His seizures stopped following a switch to a non-efavirenz-based regimen.

**Conclusions:**

This report brings to light the occurrence of complex partial seizures in patients on efavirenz. It also demonstrates the effective resolution of seizures when efavirenz treatment is replaced with a non-efavirenz-based regimen.

## Background

The 2010 global estimate of persons living with HIV was 33.4 million, with about 22.5 million (67 %) living in sub-Saharan Africa [1]. Highly active antiretroviral therapy (HAART) is essential in the reduction of morbidity and mortality related to HIV infection. It improves the quality of life and life expectancy of individuals with HIV infection. HAART results in sustained suppression of plasma HIV viral load and a significant improvement in immune status as measured by absolute and percentage cluster of differentiation 4-positive (CD4^+^) counts [2, 3]. Efavirenz is a commonly prescribed antiretroviral drug.

Although adherence to antiretroviral drug therapies is invaluable in the successful management of patients with HIV [4], adverse drug reactions are a major factor militating against use of these drugs [5]. Efavirenz frequently causes central nervous system (CNS) symptoms such as dizziness and hallucinations [6]; it can also precipitate psychosis, especially in patients with a previous history of psychiatric illness [7]. Seizure disorder is a documented side effect of efavirenz [8]. There are some case reports of efavirenz causing various types of seizure disorders, such as status epilepticus and absence seizures [8, 9].

## Case presentation

A 33-year-old Nigerian man who presented with HIV infection was commenced on HAART (tenofovir 300 mg, emtricitabine 200 mg, and efavirenz 600 mg) for about 1 month and then developed generalized tonic-clonic seizures that occurred once in 3 months. He had no preceding history of seizures or family history of seizures, nor did he have any history suggestive of CNS infection or any use of psychotropic drugs or other form of drug treatment.

**Fig. 1:**
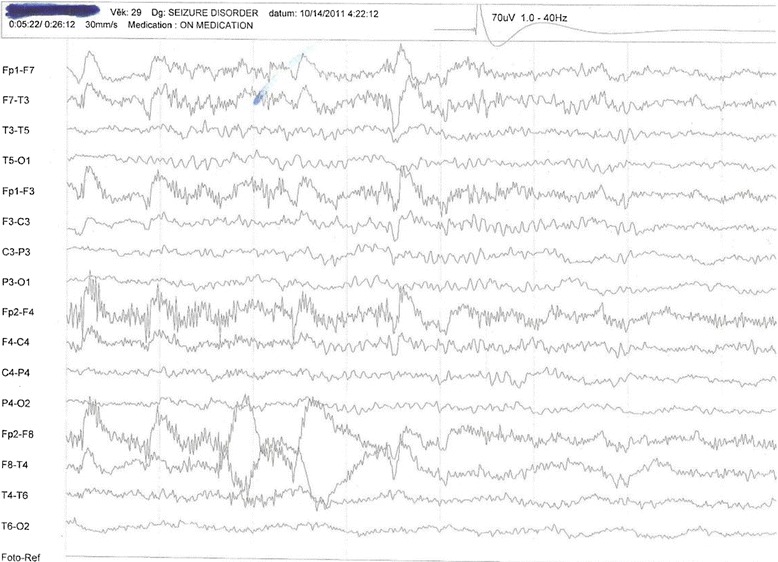
Electroencephalogram of the patient with human immunodeficiency virus while on efavirenz, showing complex partial seizures, October 2011

**Fig. 2:**
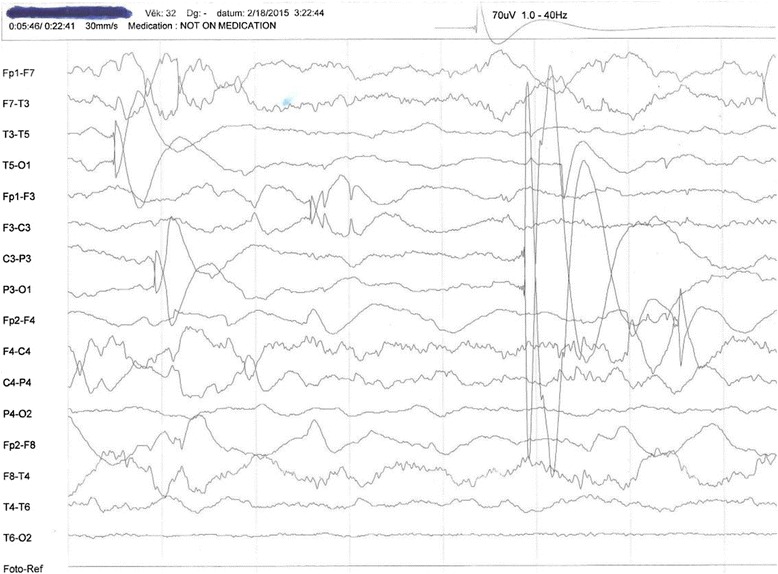
Electroencephalogram of the patient with human immunodeficiency virus when off efavirenz, showing normal EEG pattern, February 2015

His clinical examination results were normal. His blood pressure was 120/80 mmHg; his baseline CD4 count was 130 cells/mm^3^; his blood sodium and glucose levels were normal at 137 mmol/L and 5.5 mmol/L, respectively; and his urea and creatinine were also within normal limits. Although his magnetic resonance imaging findings were unremarkable, an electroencephalogram showed intermittent bursts of high-voltage sharp waves and spikes bilaterally over frontotemporoparietal regions, consistent with complex partial seizures (Fig. 1). The patient was initially administered sodium valproate with no appreciable control of seizures. His efavirenz plasma level was measured using high-performance liquid chromatography and was found to be 209.55 μg/ml. Efavirenz was subsequently replaced with nevirapine, resulting in instant resolution of his seizures. He has been seizure free for 3 years and has not required the use of any anticonvulsant. A repeat electroencephalogram showed no sign of seizure activity (Fig. 2).

## Discussion

Efavirenz readily crosses the blood-brain barrier owing to its lipophilic nature. It attains a therapeutic concentration in the cerebrospinal fluid that is about 0.5–1.2 % of its corresponding plasma level [10]. Over 50 % of individuals initiated on an efavirenz-based regimen complain of CNS-related side effects. However, these side effects are usually mild and resolve within 1 month of initiating therapy. A rare but well-documented adverse effect of efavirenz is seizure disorder. Our patient showed evidence of complex partial seizures that persisted despite intervention with an antiepileptic drug (sodium valproate). His seizures resolved following replacement of efavirenz with nevirapine in the antiretroviral regimen.

Measurement of our patient’s efavirenz plasma concentration revealed an extremely high value of 209.55 μg/ml (normal range 1–4 μg/ml) [11]. Conflicting findings have been reported regarding the association between efavirenz plasma levels and neuropsychiatric adverse events. Researchers in some studies have reported that higher efavirenz concentration predisposes patients to CNS toxicity [6, 12], whereas others have reported no relationship at all [14]. The abnormal level of efavirenz observed in our patient may have been due to racial differences in the metabolism of the drug. Patients of African origin tend to have more cytochrome P2B6 (CYP2B6) G516T and CYP2B6 T516T genotypes than their white counterparts and are regarded as poor metabolizers of efavirenz [12, 13]. People who are homozygous for the CYP2B6 516T/T genotype, which is associated with poor efavirenz clearance, are more at risk of efavirenz-induced seizure disorder. The half-life of efavirenz is 40–55 h; in addition, efavirenz is highly protein bound and may be associated with increased adverse effects, including CNS toxicity, especially when taken with food because of increased absorption [14]. The mechanism of efavirenz-induced seizure disorder is unknown; however, it may be a direct epileptogenic effect, especially at high doses or with poor drug clearance and drug-drug interaction [6]. The limitations of our present case are our inability to determine the patient’s cytochrome P450 genotype and that his blood calcium and magnesium levels were not measured.

## Conclusions

This report presents information regarding the occurrence of complex partial seizures in patients receiving efavirenz treatment. It also demonstrably posits that replacing the culpable antiretroviral drug with a non-efavirenz-based regimen can be extremely effective.

## Consent

Written informed consent was obtained from the patient for publication of this case report and any accompanying images. A copy of the written consent is available for review by the Editor-in-Chief of this journal.

## Abbreviations

CD4: cluster of differentiation 4
CNS: central nervous system
CYP2B6: cytochrome P2B6
HAART: highly active antiretroviral therapy
